# Harmonization and resulting ease with which researchers can discover and combine datasets; federated approaches

**DOI:** 10.1002/alz70858_101427

**Published:** 2025-12-25

**Authors:** Sarah Bauermeister

**Affiliations:** ^1^ Dementias Platform UK ‐ University of Oxford, Oxford, United Kingdom

## Abstract

**Background:**

Dementias Platform UK (DPUK) Data Portal is designed to facilitate access multi‐modal cohort datasets for dementias research. Data storage and analysis facilities are provided accessing >65 population and clinical cohorts (*n* = 3.7m), by >1,100 users in 44 countries. International cross‐platform collaborations are underway with the AD Workbench, Dementias Platform Australia (DPAU), and colleagues in South Korea (Korean Dementia Research Centre (KDRC), Korean Brain Research Institute (KBRI), Yonsei University).

Central to these collaborative projects are data pre‐processing pipelines using standard modality‐specific data models, data discovery and access brokerage, governance‐flexible federation models, and access to electronic health records. All of these can represent significant barriers to global data sharing. Examples of how these issues have been addressed, with relative degrees of success, will be presented with exemplar scientific collaborative projects using standardised and federation model pipelines:

**Methods:**

1. The impact of intergenerational transmission of trauma in dementia‐like neurodegeneration

2. The relationship between stages of frailty and cognitive decline

3. Childhood adversity and late‐life mental health and wellbeing

4. Mental health trajectories during the COVID‐19 pandemic

**Results:**

Project 1: Wet‐lab and dry‐lab integrative approach we are investigating intergenerational transmission of trauma in dementia across murine and human models. Preliminary results show that a structure shared in both animal and human models is implicated, the habenula (DPUK and KBRI)

Project 2: Federation model and standardised psychosocial measures (C‐Surv) assessing the associations between frailty stage and cognitive decline in ELSA UK and MAS (DPUK and DPAU)

Project 3: Federation model and standardised biopsychosocial measures (C‐Surv) assessing prediction models of early adversity and later life mental health and wellbeing. Using ELSA UK and MAS datasets, preliminary models show that early adversity predicts later life poor mental health and wellbeing (DPUK and DPAU)

Project 4: Using a federation model and harmonised psychosocial measures we apply a temporal trend analysis to multi‐site datasets (ELSA UK, ELSA Brazil, Brains for Dementia Research (BDR) and the AllOfUs study).

**Conclusions:**

Using established curation, harmonisation and federation pipelines, DPUK are working with partners to collaborate on exemplar neurodegenerative disease‐focused projects which avoid data transfer.

